# The etiology and outcomes of cardiopulmonary resuscitation in patients who are on V-V ECMO, a letter to the editor^☆^

**DOI:** 10.1016/j.resplu.2023.100536

**Published:** 2023-12-22

**Authors:** Mazen Odish, Erin Roberts, Travis Pollema, Erica Pentony, Cassia Yi, Robert L. Owens, Gabriel Wardi, Rebecca E. Sell

**Affiliations:** UC San Diego, Department of Medicine, Division of Pulmonary, Critical Care, Sleep Medicine, and Physiology, United States; UC San Diego, Department of Medicine, Division of Pulmonary, Critical Care, Sleep Medicine, and Physiology, United States; UC San Diego, Department of Surgery, Division of Cardiovascular and Thoracic Surgery, United States; UC San Diego Health, Department of Nursing, United States; UC San Diego Health, Department of Nursing, United States; UC San Diego, Department of Medicine, Division of Pulmonary, Critical Care, Sleep Medicine, and Physiology, United States; UC San Diego, Department of Medicine, Division of Pulmonary, Critical Care, Sleep Medicine, and Physiology, United States; UC San Diego, Department of Medicine, Division of Pulmonary, Critical Care, Sleep Medicine, and Physiology, United States

*To the Editor*, outcomes for hospitalized patients who suffer cardiac arrest (CA) remain poor, with 25% surviving to discharge.^1^ Surprisingly, the extracorporeal life support organization (ELSO) database reports similar survival to discharge after cardiac arrest for patients on veno-venous extracorporeal membrane oxygenation (V-V ECMO) at 23.2%, although data on etiology and neurologic outcomes are minimal.^2^ Understanding the etiologies and outcomes of CA in these patients may help inform how providers counsel patients and families and potentially identify patients at high risk of CA.

Between 2017–2021, there were 139 V-V ECMO runs at our institution. Of these, 13 patients had a CA while on V-V ECMO, **(**Table 1**).** CA was defined as lack of pulse greater than or equal to 20 seconds, a defibrillation, and/or chest compressions. The average age of our cohort was 41.5 ± 12.7 years old, all were men, and the average BMI was 31.1 ± 7.7 compared to all V-V ECMO patients with an average age of 44.9 ± 13.1, 77.5% men, and BMI of 29.5 ± 6.7. Most required V-V ECMO for viral acute respiratory distress syndrome (ARDS) (n = 8), while the rest were awaiting lung transplant (n = 3) or being treated for asthma exacerbation (n = 1) or bacterial pneumonia (n = 1). Four patients (31%) were on continuous renal replacement therapy during the cardiac arrest.^2^, ^3^, ^4^ CA was precipitated by hypoxemia (n = 8, 4 occurring during an oxygenator exchange), hypotension (n = 3), ECMO loss of battery power (n = 1), and brain herniation (n = 1), with an initial arrest rhythm of pulseless electrical activity (PEA) (n = 8), asystole (n = 4), and ventricular fibrillation (n = 1). The average length of cardiopulmonary resuscitation (CPR) was 3.3 ± 3.1 minutes with 100% (13/13) achieving return of spontaneous circulation (ROSC), and 23% (n = 3) survived to hospital discharge. There was no ECMO circuit complications post-CA such as fractured or mispositioned cannulas. All 3 survivors had a cerebral performance category of 1 at discharge. Overall survival of the 139 V-V ECMO patients from 2017-2021 was 57.5%.

**Table 1:** Patient demographics, ECMO data, arrest characteristics, and outcomes. ARDS, acute respiratory distress syndrome. Venous insufficiency is defined as an ECMO circuit that is unable to provide an adequate blood flow rate due to a lack of venous return from the drainage cannula.

**Patient**	**Age, years**	**Sex**	**BMI, kg/m^2^**	**Indication for V-V ECMO**	**Days on ECMO until Cardiac Arrest**	**SOFA Score Pre-arrest**	**Etiology of Cardiac Arrest**	**Initial Cardiac Arrest Rhythm**	**Length of Cardiac Arrest, minutes**	**ROSC Achieved**	**Total ECMO Days**	**Total Hospital Days**	**Cerebral Vascular Complications post Cardiac Arrest**	**Survival to Hospital Discharge**
**1**	46	M	27.1	Bridge to lung transplant from ILD	13	7	Hypoxemia during oxygenator exchange	PEA	2	Yes	17	32	No	No
**2**	21	M	18.7	Bridge to lung transplant from CF	5	10	Hypoxemia during oxygenator exchange	Asystole	7	Yes	8	21	No	No
**3**	33	M	40.6	Asthma Exacerbation	6	6	Brain herniation (VF)	VF	2	Yes	9	20	Yes	No
**4**	64	M	21.4	Bacterial pneumonia post Esophagectomy	5	18	Hypotension	Asystole	0.67	Yes	11	89	Yes	No
**5**	19	M	20.8	Bridge to lung transplant from CF	17	9	Hypoxemia from venous insufficiency	PEA	5	Yes	19	20	Yes	No
**6**	39	M	38.8	ARDS from Adenovirus	7	15	Hypoxemia from venous insufficiency	PEA	2	Yes	11	48	No	Yes, discharged to SNF
**7**	49	M	29	ARDS from COVID	28	8	Hypoxemia	PEA	2	Yes	69	85	Yes	No
**8**	56	M	33.9	ARDS from COVID	13	11	Hypotension	PEA	0.5	Yes	25	34	No	No
**9**	47	M	31	ARDS from COVID	15	8	V-V ECMO machine failure (battery power loss)	Asystole	5	Yes	36	44	No	No
**10**	35	M	43.7	ARDS from COVID	49	7	Hypoxemia during oxygenator exchange	PEA	0.5	Yes	59	60	No	No
**11**	45	M	35.8	ARDS from COVID	2	9	Hypotension	PEA	11	Yes	16	29	No	No
**12**	48	M	31.9	ARDS from COVID	130	5	Hypoxemia during oxygenator exchange	PEA	5	Yes	224	266	No	Yes, lung transplant, discharged home
**13**	37	M	31.4	ARDS from COVID	42	6	Hypoxemia from venous insufficiency	Asystole	0.5	Yes	60	77	No	Yes, discharged to LTAC
**Average** ± **SD** **(Percent)** **Median (IQR)**	41.1 ± 12.7 13 (19–64)		31.1 ±7.7 31.5 (27.1––35.8)		25.5 ± 34.6 13 (6–28)	9.2 ± 3.7 8 (7–10)			3.3 ± 3.1 2 (0.6–5)	13/13 (100%)	43.4 ± 58.3 19 (11–59)	63.5 ± 65.6 44 (29–77)	4/13 (30.7%)	3 (23.1)

BMI, body mass index. CF, cystic fibrosis. COVID, Coronavirus disease of 2019. ILD, interstitial lung disease. ROSC, return of spontaneous circulation. SOFA, sequential organ failure assessment. PEA, pulseless electrical activity. VF, ventricular fibrillation. V-V ECMO, veno-venous extracorporeal membrane oxygenation. IQR, interquartile range. SNF, skilled nursing facility. LTAC, long term acute care.

With time, ECMO oxygenators require replacement, which temporary disrupt the circuit resulting in a transient worsening of hypoxemia. During oxygenator exchanges at our institutional, it is standard to have the primary team present, resuscitation cart, maximize ventilator support, and prepare vasoactive medications for potential circulatory embarrassment. ECMO teams should exercise vigilance during oxygenator exchanges for severely hypoxemic despite V-V ECMO support.

Our data informs clinicians caring for patients on V-V ECMO about the effectiveness of CPR. While it may be controversial to offer CPR to patients with V-V ECMO, we found high ROSC rates despite presumed futility. While there is limited evidence for the effectiveness of CPR for patients on V-V ECMO, in this small sample, ROSC was universal, and 23% of patients survived to discharge with a CPC score of 1, similar to other CA cohorts.^1^ Our findings need to be validated at other ECMO centers although we note similar rates by Brooke et al.^5^ Future studies should continue to study the utility of CPR on V-V ECMO and how to minimize circuit disruptions (i.e., oxygenator exchanges and venous insufficiency) that may result in CA.

## Declaration of competing interest

The authors declare that they have no known competing financial interests or personal relationships that could have appeared to influence the work reported in this paper.
